# Predicting Positive p53 Cancer Rescue Regions Using Most Informative Positive (MIP) Active Learning

**DOI:** 10.1371/journal.pcbi.1000498

**Published:** 2009-09-04

**Authors:** Samuel A. Danziger, Roberta Baronio, Lydia Ho, Linda Hall, Kirsty Salmon, G. Wesley Hatfield, Peter Kaiser, Richard H. Lathrop

**Affiliations:** 1 Institute for Genomics and Bioinformatics, University of California, Irvine, Irvine, California, United States of America; 2 Department of Biomedical Engineering, University of California, Irvine, Irvine, California, United States of America; 3 Department of Microbiology and Molecular Genetics, University of California, Irvine, Irvine, California, United States of America; 4 Department of Biological Chemistry, University of California, Irvine, Irvine, California, United States of America; 5 Department of Computer Science, University of California, Irvine, Irvine, California, United States of America; University of Houston, United States of America

## Abstract

Many protein engineering problems involve finding mutations that produce proteins with a particular function. Computational active learning is an attractive approach to discover desired biological activities. Traditional active learning techniques have been optimized to iteratively improve classifier accuracy, not to quickly discover biologically significant results. We report here a novel active learning technique, Most Informative Positive (MIP), which is tailored to biological problems because it seeks novel and informative positive results. MIP active learning differs from traditional active learning methods in two ways: (1) it preferentially seeks Positive (functionally active) examples; and (2) it may be effectively extended to select gene regions suitable for high throughput combinatorial mutagenesis. We applied MIP to discover mutations in the tumor suppressor protein p53 that reactivate mutated p53 found in human cancers. This is an important biomedical goal because *p53* mutants have been implicated in half of all human cancers, and restoring active p53 in tumors leads to tumor regression. MIP found Positive (cancer rescue) p53 mutants *in silico* using 33% fewer experiments than traditional non-MIP active learning, with only a minor decrease in classifier accuracy. Applying MIP to *in vivo* experimentation yielded immediate Positive results. Ten different p53 mutations found in human cancers were paired *in silico* with all possible single amino acid rescue mutations, from which MIP was used to select a Positive Region predicted to be enriched for p53 cancer rescue mutants. *In vivo* assays showed that the predicted Positive Region: (1) had significantly more (p<0.01) new strong cancer rescue mutants than control regions (Negative, and non-MIP active learning); (2) had slightly more new strong cancer rescue mutants than an Expert region selected for purely biological considerations; and (3) rescued for the first time the previously unrescuable p53 cancer mutant P152L.

## Author Summary

Engineering proteins to acquire or enhance a particular useful function is at the core of many biomedical problems. This paper presents Most Informative Positive (MIP) active learning, a novel integrated computational/biological approach designed to help guide biological discovery of novel and informative positive mutants. A classifier, together with modeled structure-based features, helps guide biological experiments and so accelerates protein engineering studies. MIP reduces the number of expensive biological experiments needed to achieve novel and informative positive results. We used the MIP method to discover novel p53 cancer rescue mutants. p53 is a tumor suppressor protein, and destructive p53 mutations have been implicated in half of all human cancers. Second-site cancer rescue mutations restore p53 activity and eventually may facilitate rational design of better cancer drugs. This paper shows that, even in the first round of in vivo experiments, MIP significantly increased the discovery rate of novel and informative positive mutants.

## Introduction

Engineering existing proteins to change their properties [1],[2] is an important task with many applications as diverse as environmental protection, synthetic biomaterials, and pharmacology [3]–[8]. Here we apply machine learning techniques to engineer the tumor suppressor protein p53. We choose where to mutate cancerous p53 to restore tumor suppressor function, using structure-based features derived from *in silico* protein homology models.

### Biology of p53 Cancer Rescue Mutants

The *p53* gene encodes a tumor suppressor protein that is a key cellular defense against cancer. p53 mutations occur in about 50% of human cancers. The vast majority of these mutations are single point missense mutations in the *p53* core domain [9]–[12]. Thus, many human cancers express full-length p53 cancer mutants that lack tumor suppressor function. As demonstrated *in vivo*, p53 cancer mutants can be reactivated through intragenic second-site suppressor (“cancer rescue”) mutations [13]–[15]. Reactivated p53 holds great therapeutic promise because animal models have shown that reintroduction of active p53, even in advanced tumors, leads to tumor regression [16]–[18]. Consequently, there have been many efforts to find small molecule drugs that mimic the cancer rescue effect of reactivating p53 and suppressing tumor growth [19]–[24]. Despite some promising discoveries in p53 in specific, and small molecule docking in general, these efforts are hampered by a limited understanding of the p53 mutation-structure-function relationship [11], [25]–[28]. A larger and more diverse collection of cancer rescue mutations that reactivate p53 cancer mutants is therefore desired. Such a collection could lead to insight into general structural changes that can rescue p53 cancer mutants, and thereby facilitate rational drug design approaches by exploiting similar effects.

Several p53 cancer rescue mutants were identified previously by random mutagenesis in a region spanning amino acid residues 225–241. A portion of this region (235,239, and 240) thus was empirically identified as a “Global Suppressor Motif”, the first p53 cancer rescue region [13]. The biological goal of this paper is to use computational techniques to discover novel p53 cancer rescue mutants and regions.

### Integrated Experimental Design

The active learning paradigm was developed in the machine learning community to reduce the number of expensive examples that need to be acquired to build an accurate classifier [29]. Active learning typically starts with a small initial amount of labeled data. The initial data is used to determine a small informative set of unlabeled examples to label. Once labeled, these new examples are added to the pool of labeled data and a new unlabeled set is chosen. The process repeatedly labels new data until the classifier reaches some pre-determined criteria. Active learning methods increase the efficiency and cost effectiveness of the process by reducing the number of examples that need to be labeled. The active learning paradigm is readily applicable to biological experimentation, as it reduces the number of tedious and expensive experiments to be performed.

In a biological active learning paradigm, a computational classifier is trained with an initial set of examples labeled by direct experimentation. In the case of p53 cancer rescue mutants [4], this initial set consists of empirically labeled p53 mutants. The computational classifier then predicts which mutants should next be labeled to most improve the classifier accuracy. These mutants are then made, labeled by biological assays, and added to the classifier. The cycle repeats, iteratively improving classifier accuracy and adding to the set of p53 mutants with known function. In this way, an optimum active learning classifier would adequately explore a mutant sequence space while using a minimum amount of expensive biological experimentation [4].

It is important to note that in the context of biological experimentation, the slowest part of active learning is generally the biological experiments required to label the unknown examples. Therefore, any reference to speed in this paper refers to the number of expensive biological experiments (i.e. iterations of the active learning cycle) and not to computational speed. The computational goal of this paper is to provide and test computational methods that can discover gene regions wherein mutations produce proteins with a desired function, while requiring as few experiments as possible.

### Traditional Active Learning

Here we present a formal description of the active learning problem. Notation is summarized in Text S1.

Let 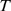 be the Total set of all examples under consideration. Each example mutant, 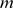, has a labeling function, 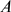, such that 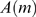 = Positive, Negative, or Unknown. During each active learning iteration, 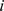, 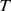 is partitioned into two groups: (1) 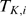, examples with Known labels (i.e., 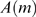 = Positive or Negative); and (2) 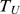, examples with Unknown labels (i.e., 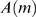 = Unknown). A third set, 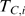, Chosen from 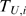, contains 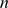 examples to be tested and labeled in this step.

During each iteration the classifier provides a decision function, 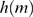, trained on the examples with a known label, 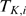. Each unlabeled example 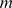 is predicted by the decision function 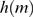 to be Positive or Negative.

A score function, 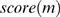, ranks each example in 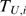. As a control, Random active learning assigns each mutant a random score. The 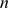 highest ranked examples become 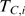 and are then tested and labeled. 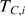
is merged with 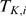 to create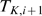 and deleted from 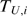
to create 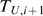.

In the case of the p53 cancer rescue mutants here, each example 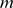
is a p53 mutant. 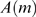 = Positive if and only if mutant 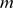
exhibits wild-type like p53 transcription activator activity.

### Structure of this Paper

The Methods section presents a description of active learning, the MIP paradigm, computational evaluation methods, and the biological experimental design. The Results section shows *in silico* results indicating the computational techniques best suited to the p53 cancer rescue mutant problem and *in vivo* results showing how well those techniques performed in experiments. The Discussion section recites medical significance, sketches possible computational extensions of the MIP method, and concludes that a computational classifier and modeled structure-based features can guide function-based experimental discovery.

## Methods

Active learning refers to a body of iterative machine learning techniques designed to train an accurate classifier using the minimum number of expensive examples [29]–[32]. The Most Informative Positive (MIP) method, introduced here, preferentially selects examples (i.e., p53 mutants) predicted to be both informative and Positive. The MIP computational method can be used to modify any active learning method that does not consider predicted class as a criterion for choosing which expensive examples to learn. Here, MIP modified the active learning algorithms described in [4] and was used to select regions in the p53 tumor suppressor protein.

This section contains:

1. An introduction to structure-based features and active learning.
2. A description of the MIP active learning method.
3. Metrics for evaluating how quickly an active learning algorithm uncovers Positive mutants.
4. The data sets used for *in silico* evaluation.
5. The general Regional Selection algorithm.
6. Regional Selection as implemented for the p53 cancer rescue problem.
7. A brief overview of the biological techniques used to test the mutant regions.

### Foundations: Structure-Based Features and Active Learning

The techniques presented in this paper build on previous research using machine learning techniques to find p53 cancer rescue mutants [3],[4]. This section contains a brief overview of the foundational structure-based features and active learning techniques.

Structure-based features [3],[4] for each mutant considered were extracted from atomic-level homology models. Modeled mutant proteins were produced *in silico* using the B chain of the wildtype p53 core domain crystal structure (PDB ID: 1TSR) [33]. Amino acids were substituted and model energies were minimized using the Amber™ molecular modeling software [34]. Features [3] were extracted from the minimized mutant model using 1D sequence and amino acid substitution information, 2D surface cartographical and electrostatic models, 3D steric analysis, and “4D” thermal stability predictions. Those features on the surface of the p53 core domain outside known binding sites [35] were compressed, resulting in 5,867 features per mutant. Conditional Mutual Information Maximization [36] selected various subsets of these features. It was found that 550 selected features resulted in the highest classifier accuracy [4].

Seven previously studied [4] active learning algorithms were used here. Two of these methods are standard active learning techniques, taken from the literature, that work by separating the data into two classes with an n-dimensional hyper-plane. Minimum Marginal Hyperplane [37] selects examples based on the margin, i.e., the “distance” from the hyper-plane. Maximum Entropy [38] selects examples based on a class probability calculated from the margin and is related to the information theory concept of entropy. Two methods, Maximum Marginal Hyperplane and Minimum Entropy, are negative controls expected to perform badly. They were created by choosing the least informative example in the previous two methods. The other three methods were created specifically for this p53 cancer rescue research project [4] and are based on the anticipated change in classifier accuracy or correlation coefficient if a given example is chosen and labeled. These include Additive/Maximum Curiosity [4], which uses a cross-validated correlation coefficient to estimate classifier accuracy, and Additive Bayesian Surprise, which is based on the Kullback-Leibler (KL) divergence [39].

### MIP Methodology

MIP optimizes the mutants chosen so that they are most likely to both improve the classifier and rapidly uncover Positive examples. To understand why this is important, suppose that Positive examples are sparse, as here, and one has only sufficient resources to assay 100 new examples. MIP active learning seeks to maximize the number of novel Positive examples discovered during those 100 assays, and at the same time quickly improve classifier accuracy. Traditional active learning also seeks to find an accurate classifier quickly, but may discover only very few novel Positives while so doing.

MIP active learning chooses 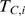 by first considering only those unlabeled examples predicted to be Positive (i.e., 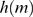 = Positive). Those predicted to be Positive and having the highest score, 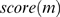, are chosen for 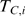. Only if too few examples in 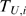 were predicted to be Positive would a Negative informative example be chosen for 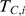.

Active learning algorithms may become MIP algorithms by preferentially labeling those informative examples that are also predicted to be Positive. There are many ways to apply MIP to a specific active learning algorithm. Here we give a simple example, which shows a general approach and applies to nearly all active learning algorithms. Recall that 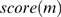 ranks unlabeled examples, and high-ranking examples are chosen to be labeled at the next iteration. To convert a traditional active learning algorithm to a MIP active learning algorithm, it is sufficient to weight the scoring function so that examples predicted to be Positive have a higher score than those predicted to be Negative:
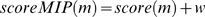
(1)where 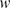 is a constant with 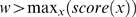 if 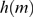 = Positive, and 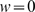 if 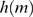 = Negative.

### Metrics: Halfway Point, Accuracy, Correlation Coefficient

For this paper and much biological research, the goal of iterative exploration is to uncover as many informative Positive examples as quickly as possible, i.e., with the fewest biological experiments. We require metrics to measure success at this task.

The Halfway Point metric measures the fraction of iterations necessary before half of all Positive examples in an unlabeled data set are uncovered. Several additional metrics were explored to measure how quickly Positive examples were found, including enrichment factor and positive area, but only Halfway Point is presented here for illustrative clarity because it is simple to explain and it provides similar results to the other metrics.

Formally, Halfway Point = 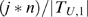, where 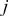
is the smallest number of iterations such that 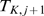
contains half of all Positive mutants in 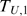 and 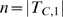
is the number of mutants labeled at each iteration.

Since MIP optimizes a classifier to preferentially choose Positive mutants for 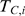, it is reasonable to wonder if there may be a corresponding loss of classifier accuracy. One way to estimate classifier accuracy for an active learning algorithm is to use the average 10-fold cross-validated accuracy and correlation coefficient of the training set 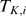 across all iterations of one or more of the Data Partitions described below. Accuracy is the fraction of correct predictions. The correlation coefficient is a standard of the machine learning community [40], and a better measure than accuracy when the data set contains unbalanced numbers of Positive and Negative examples. This is the usual case for biological data sets such as the p53 cancer rescue mutant data set, where the ratio of Negative to Positive mutants is about 4∶1.

Several other metrics for accuracy were explored, including forward prediction accuracy, 3-point accuracy, and a more complicated cross-validation strategy, OECV [4]. Average 10-fold cross-validated accuracy and correlation coefficient were chosen for illustrative clarity here because they are simple to explain and give similar results to the other metrics.

### Evaluation *In Silico*

To evaluate the MIP methodology *in silico*, MIP and non-MIP versions of seven active learning methods plus a random control were compared using the cross-validated metrics described above. Three previously studied partitions of the data set [4] were used to compare to previous research. These partitions test three common starting conditions for active learning:

1. Data Partition 1: Few mutants in 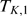
and many in 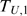, i.e., 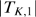 = 25 and 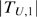 = 236.
2. Data Partition 2: Similar numbers of mutants in 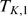
and 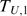, i.e., 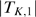 = 123 and 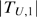 = 138.
3. Data Partition 3: Many mutants in 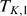
and few in 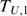, i.e., 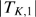 = 204 and 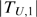 = 57.

The data set had about 20% Positive and 80% Negative mutants.

### Regional Selection

Active learning and MIP as discussed so far apply to individual mutants. Limitations of this approach include loss of classifier accuracy when applied to new mutants from unfamiliar regions, leading to many experiments that yielded few Positive examples [4]. We generalized MIP active learning to apply to single amino acid changes in contiguous gene regions. This generalization supported several desirable outcomes. It allowed MIP active learning to exploit high throughput saturation mutagenesis techniques. The resulting training set enrichment should allow more accurate prediction of new Positive mutants, especially those requiring multiple amino acid changes. Regions enriched for rescue mutants may indicate promising candidate drug target sites.

Formally, let 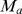 be the set of all mutants containing a cancer mutation plus a single putative rescue at amino acid location 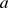, excluding mutants that exist in the initial training set 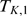. Let
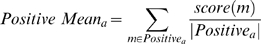
(2)where 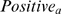 is the subset of 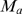
for which 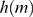 = Positive. Positive regions were ranked by summing 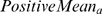 across each region. The Positive Region used below was chosen to be the 10 sequential amino acid long window with the highest average 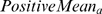 across that window.

Similarly, let
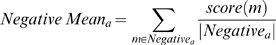
(3)where 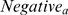
is the subset of 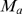
for which 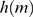 = Negative. The Negative Region was chosen to be the 10 sequential amino acid long window with the highest average 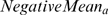 across that window.

A similar non-MIP control region was constructed to be informative to the classifier regardless of whether mutants were predicted to be Positive or Negative. Let
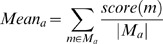
(4)


The non-MIP Region was chosen to be the 10 sequential amino acid long window with the highest average 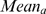 across that window.

### Regional Selection Implementation

To detect p53 cancer rescue regions, the task is to identify areas of the p53 core domain that are likely to have many Positive cancer rescue mutants. We considered ten p53 cancer mutants that are commonly found in human cancer [12] and can be constructed so that they differ by two or more nucleic acid changes from the wild-type. 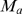 consisted of these 10 common p53 cancer mutants paired with all possible single amino acid changes at each location in the core domain. All predictions and curiosity calculations were made with a training set, 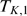, of 463 mutants (91 Positive and 372 Negative). These 463 mutants contained the 261 mutants used for the Data Partitions plus 202 created during other experiments using variants of the yeast assay described below [3],[4],[13],[14].

The MIP Additive Curiosity [4] algorithm was used to choose the regions because it performed best in *in silico* trials (see Results). It was adapted to select three 10-amino acid long regions in the p53 core domain: a Positive region, a Negative region, and a non-MIP control region. A Weka Support Vector Machine, SMO, [41], was used to predict the activity, 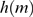, for each mutant. The score for each mutant was calculated using MIP Additive Curiosity. These values were averaged over every possible 10-amino acid window. The classifier considered the resulting 34,776 putative cancer rescue mutants and selected ∼3,980 mutants in three regions. These regions were selected for the following criteria as described above:

1. Positive Region: predicted to be informative and contain novel Positive mutants.
2. Negative Region: a control predicted to be informative and contain few Positive mutants.
3. non-MIP Region: a control predicted to be informative regardless of mutant activity.
  As another control, these regions were compared to:
4. Expert Region: a control selected for biological considerations by an expert p53 cancer rescue biologist and hypothesized to contain Positive cancer rescue mutants.

The Expert Region, spanning residues 114–123, was considered a potential cancer rescue region because several Positive mutations with multiple amino acid changes occurred there spontaneously in previous cancer rescue mutant screens. Therefore, this region was considered likely to have cancer rescue mutants with single amino acid changes ([13]; Brachmann, R. K., personal communication).

No single amino acid change cancer rescue mutations had been found previously in any of the Positive, Negative, non-MIP, or Expert regions.

### Regional Saturation Mutagenesis and Yeast Assay

All mutants produced in this study were initially created with a novel regional saturation mutagenesis method based on the Quick Change site-directed mutagenesis kit (Stratagene, La Jolla, CA, USA), (manuscript in preparation). Briefly, a set of overlapping degenerate oligonucleotides was designed such that each primer contained exactly one random codon. A standard site-directed mutagenesis reaction was performed with a mixture of oligonucleotides that collectively represented each possible codon change in the target region (30 base pairs). The overlapping primer design prevented multiple mutations in the same mutagenesis product. The generated mutants were analyzed for p53 activity using a yeast-based p53 activity assay [13].

Briefly, yeast cells were engineered to depend on active p53 for expression of the *URA3* gene. The *URA3* gene product is required for the synthesis of uracil. When cells are grown in medium lacking uracil, cell growth is proportional to p53 activity (*URA3* expression). The products of the saturation mutagenesis for all ten p53 cancer mutants in all tested regions were first selected for their ability to grow in the absence of uracil, indicating re-activated p53. All putative positive mutants were analyzed by DNA sequencing to determine the nature of the rescue mutation. Mutations were then recreated by site-directed mutagenesis, confirmed by resequencing, and retested.

**Figure 1:**
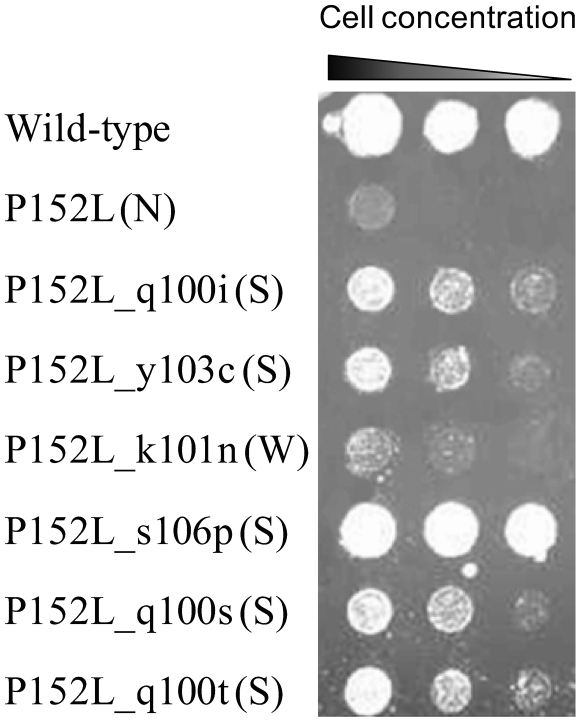
Growth results at different yeast concentrations. Wild-type refers to yeasts containing the wild-type p53 strain. Mutants annotated with (S) are strong Positive cancer rescue mutants, (W) are weak Positive cancer rescue mutants, and (N) are Negative mutants. Different numbers of yeast cells expressing wild-type or mutant p53 as indicated were spotted on growth media. The numbers of cells spotted (from left to right) was 10,000, 2,000 and 400 cells. Cells were then cultured at 37°C for 2 days and cell growth was assessed by the observable increase in cells, which is proportional to the starting cell number. Rescue mutants were designated as “strong” if they showed better growth at the 2,000 cells per spot position than the cancer mutant at 10,000 cells per spot. Rescue mutants were considered “weak” when growth advantage was only obvious when the 10,000 cells per spot were compared between rescue mutant and cancer mutant.

As shown in Figure 1, mutants were designated as strong Positive mutants if the yeast cell growth was very robust. Mutants contained in yeasts that showed minimal growth were designated as weak Positive mutants. Strong and weak Positive mutants were collectively designated Positive. Those that did not grow were designated Negative. p53 mutants are described as <Cancer Mutation>_<putative rescue mutation>. For example, P152L_q100i identifies a cancer mutation with leucine replacing proline at amino acid 152 and a putative rescue mutation with isoleucine replacing glutamine at amino acid 100.

## Results

Most Informative Positive (MIP) active learning was designed to find Positive examples, here p53 cancer rescue mutants, as quickly as possible. Fourteen active learning methods (seven implemented as MIP algorithms) and one random control were tested. The MIP method Additive Curiosity performed best *in silico*, so was used to select the Positive, Negative, and non-MIP regions. These regions were assayed for novel p53 cancer rescue mutants.

This section contains:

1. The *in silico* performance comparison of MIP and non-MIP active learning algorithms.
2. The regions selected by the regional selection algorithms.
3. Novel rescue mutants discovered in the Positive, Negative, and non-MIP regions.
4. Other predicted p53 regions.
5. 3D Visualizations of the putative rescue regions and significant mutants.

### Comparison of MIP and non-MIP Active Learning Methods

For the purposes of this study, the best active learning method was the method with the lowest Halfway Point, i.e., the method that discovered half of the Positive mutants in the test set using the smallest fraction of possible iterations. From Table 1, the best MIP method reached the Halfway Point in 33% fewer iterations, and the average MIP algorithm needed 28% fewer iterations, than their non-MIP counterparts (p<0.006). Even the MIP versions of the negative control methods, Maximum Marginal Hyperplane and Minimum Entropy, performed better than any of the non-MIP methods.

**Table 1:** Active learning halfway points.

Active Learning Type	non-MIP	MIP
**Additive Curiosity**	0.472	**0.317**
**Maximum Curiosity**	**0.406**	0.341
**Minimum Marginal Hyperplane**	0.423	0.356
**Additive Bayesian Surprise**	0.463	0.365
**Maximum Entropy**	0.461	0.388
**Minimum Entropy**	0.666	0.381
**Maximum Marginal Hyperplane**	0.639	0.403
**Random (100 Trials)**	0.502	+/−0.084

The Halfway Points are averaged across the three data sets discussed in the Methods section. Applying a paired Student's t-test to these seven active learning methods reveals a two-tailed p-value = 0.011.

**Figure 2:**
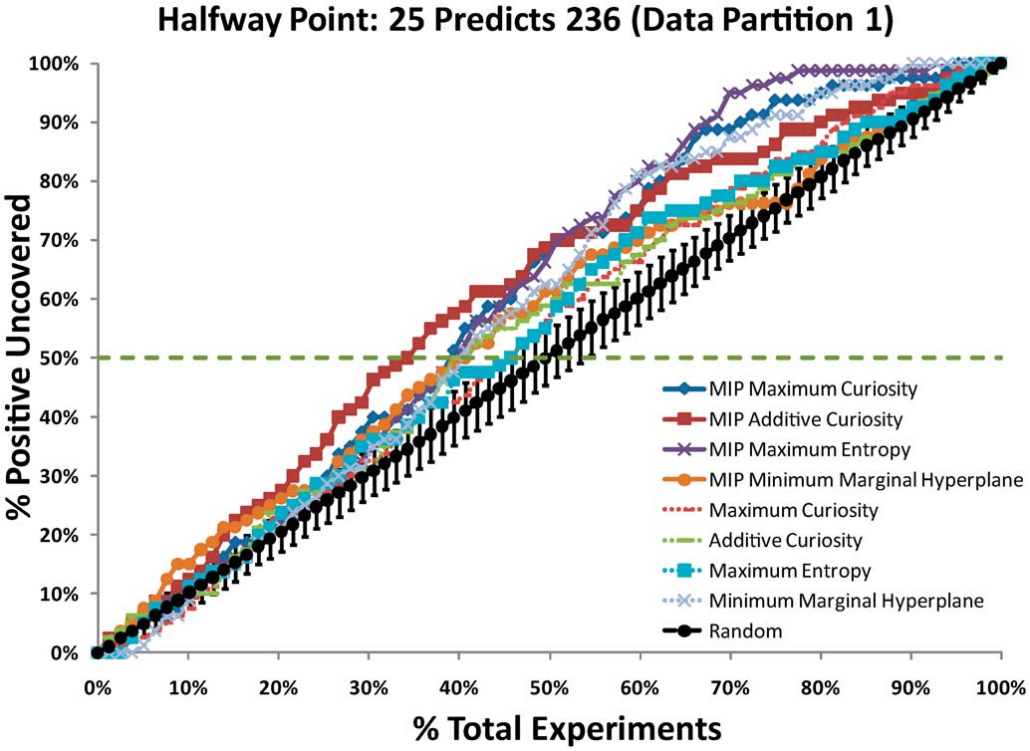
MIP versus non-MIP halfway points. Shown are the fraction of Positive mutants uncovered by MIP Maximum Curiosity and Additive Curiosity compared with their non-MIP counterparts. The intersections with the dotted horizontal line correspond to the Halfway Point.

A graph showing the Halfway Point for select active learning types with Data Partition 1, 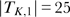 = 25 and 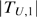 = 236, is presented in Figure 2.

Applying the MIP methodology improves how quickly a given active learning algorithm uncovers the Positive mutants, but what effect does it have on overall classifier accuracy? The 10-fold cross-validated results, presented in Table 2 and Table 3, show that MIP reduced the cross-validated accuracy by on average 1.1% (statistically significant, p-Value = 0.012) and the correlation coefficient by on average 0.001 (not significant, p-Value = 0.755).

**Table 2:** 10-fold cross-validated accuracy.

Active Learning Type	non-MIP	MIP
**Additive Curiosity**	73.4%	**73.5%**
**Maximum Curiosity**	72.9%	72.7%
**Minimum Marginal Hyperplane**	73.4%	72.1%
**Additive Bayesian Surprise**	74.7%	72.5%
**Maximum Entropy**	73.3%	72.1%
**Minimum Entropy**	73.4%	72.3%
**Maximum Marginal Hyperplane**	**74.9%**	73.1%
**Average of seven methods above**	73.7%	72.6%
**Random (100 Trials)**	72.4%	+/−4.36

The average 10-fold cross-validated accuracy for all training sets across the three Data Partitions discussed in the Methods section. Applying a paired Student's t-test to these seven active learning methods reveals a two-tailed p-value = 0.012.

**Table 3:** 10-fold cross-validated correlation coefficient.

Active Learning Type	non-MIP	MIP
**Additive Curiosity**	.402	**.423**
**Maximum Curiosity**	.390	.400
**Minimum Marginal Hyperplane**	.404	.392
**Additive Bayesian Surprise**	**.428**	.404
**Maximum Entropy**	.393	.386
**Minimum Entropy**	.370	.381
**Maximum Marginal Hyperplane**	.409	.396
**Average of seven methods above**	.399	.398
**Random (100 Trials)**	.304	+/−.131

The average 10-fold cross-validated correlation coefficient for all training sets across the three Data Partitions discussed in the Methods section. Applying a paired Student's t-test to these seven active learning methods reveals a two-tailed p-value = 0.755.

### Positive, Negative, Non-MIP, and Expert Regions

**Figure 3:**
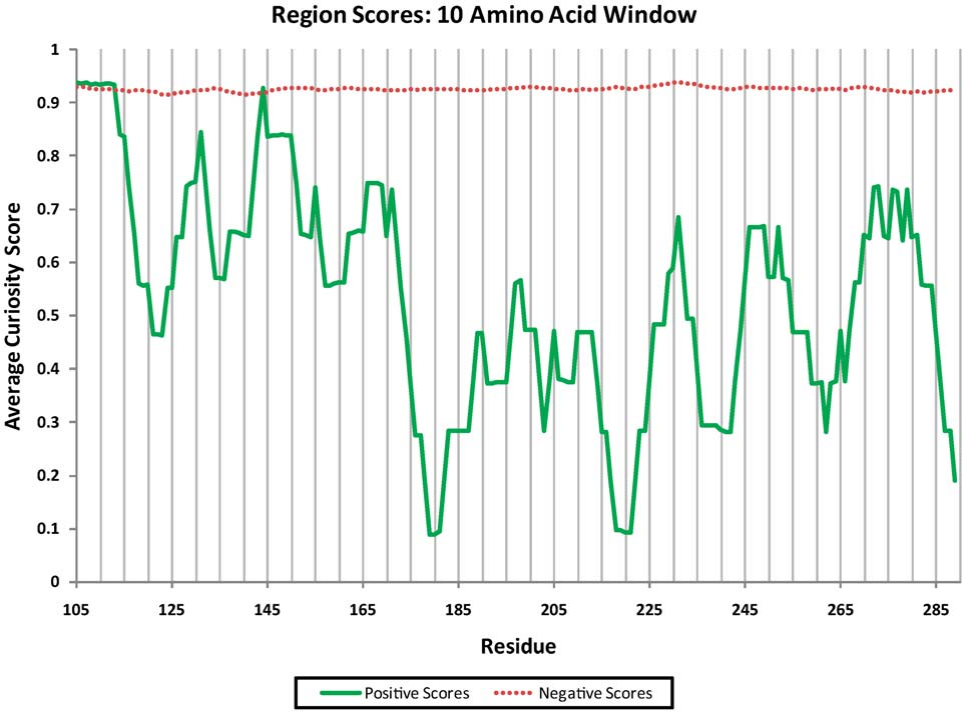
Scores for Positive and Negative Regions using Additive Curiosity. The score at each residue is the average Additive Curiosity score for the preceding ten residues. For example, the Positive Score at residue 105 scores the region from 96–105 to test if it is the best Positive Region. The non-MIP Scores are omitted because they are nearly indistinguishable from the Negative Scores.

**Figure 4:**
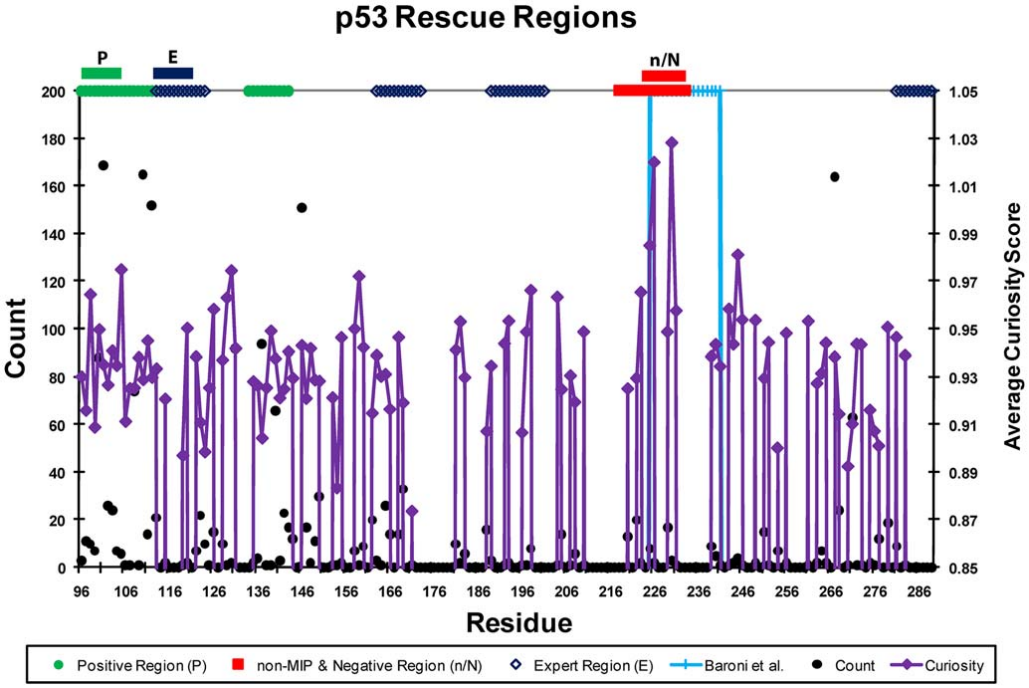
Regional saturation mutagenesis scores and selections. Count indicates the number of mutants predicted to be Positive at each residue in the p53 core domain and is represented as black dots corresponding to the leftmost y-axis. Average Curiosity Score is the average Additive Curiosity score for the mutants predicted to be Positive at each residue and is represented as solid purple diamonds connected with lines and corresponding to the rightmost y-axis. The solid green circles indicate contiguous regions of 10 or more residues that have high Curiosity and are predicted to be Positive. The solid red squares indicate the contiguous regions of 10 or more residues that have high Curiosity and are predicted to be Negative. The purple diamonds indicate contiguous regions that an expert might expect to contain rescue mutants based on previous experiments. The light blue +s with the lines descending to the x-axis indicate the region explored in Baroni, et al., (2004), though this region is not treated specially, nor is even known, by the classifier. The Positive, non-MIP, Negative, and Expert regions ultimately selected are presented above the plot and labeled with P, n, N, and E respectively. No single amino acid rescue mutations had been found previously in any of the Positive, non-MIP, Negative, or Expert regions.

The MIP Additive Curiosity algorithm performed best in Tables 1, 2, and 3, and so was used to select three 10 amino acid long regions as the Positive, Negative, and non-MIP Regions. The Positive Region from residues 96–105 had the highest average 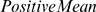 score (.938) and contained 351 mutants predicted to be Positive out of 1900 total. The Negative Region from residues 223–232 had the highest average 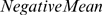 score (.937) and contained 33 mutants predicted to be Positive. The non-MIP Region from residues 222–231 had the highest 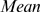 score (.938) and contained 53 mutants predicted to be Positive. For comparison, the Expert Region from residues 114–123 had a 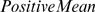 score of (.462) and contained 34 mutants predicted to be Positive. See Figure 3 for the scores across possible Positive and Negative Regions and Figure 4 for a graph illustrating those regions within the p53 core domain.

Regional Saturation Mutagenesis produced all possible single amino acid mutations in these regions combined with the 10 common cancer mutants tested. A biological selection was performed to identify all rescue mutants based on re-activation of p53 cancer mutants *in vivo*.

The summary of these results is recorded in Table 4. The Positive Region contained 8 strong and 3 weak mutants, the Expert Regions contained 6 strong and 7 weak mutants, while the Negative and non-MIP regions each contained only 2 weak mutants.

**Table 4:** Novel Positive cancer rescue mutant counts & statistics.

	Positive	Negative	non-MIP	Expert
	(96–105)	(223–232)	(222–231)	(114–123)
**# Strong**	**8**	0	0	6
p-value	-	0.008	0.008	0.791
**# Weak**	3	2	2	**7**
p-value	-	1.000	1.000	0.344
**# Total**	11	2	2	**13**
p-value	-	0.022	0.022	0.839

The range of numbers listed below the region names are the amino acid locations covered by that region. All p-values are two-tailed p-values; the corresponding one-tailed values are half what is listed.

Table 4 also shows the p-values associated with the null hypothesis “Positive mutants are equally likely to be drawn from the Positive Region as the Negative, non-MIP, or Expert Region.” From this analysis we are at least 99.5% confident (one-tail) that the Positive Region contains more strong cancer rescue mutants than the Negative or non-MIP Region. Similarly, we infer that there is no significant difference between the number of cancer rescue mutants in the Positive and Expert regions.

### Novel p53 Cancer Rescue Mutants

The novel p53 cancer rescue mutants found in the Positive, Negative, and non-MIP regions are presented in Table 5 and summarized in Table 6. Three different cancer mutants were rescued by these regions: P152L, R158L and G245S. R158L was rescued strongly by the Positive Region, and weakly by the Negative and non-MIP regions. G245S was rescued weakly by the Negative and non-MIP regions. P152L, a previously unrescued cancer mutant, was rescued only by the Positive Region, and rescued strongly.

**Table 5:** Novel Positive cancer rescue mutants by name.

Positive Region	Negative/non-MIP Region	Artifactual Mutants
P152L_q100i	*R158L_e224p (W)*	P152L_s106p
P152L_q100s	*G245S_t231y (W)*	P152L_l137m
P152L_q100t		P152L_d207e
P152L_y103c		R158L_l201p
R158L_q100f		R158L_q100h_q104a
R158L_q100n		R158L_q100a_q104r
R158L_q100s		
R158L_q100t		
*P152L_q100a (W)*		
*P152L_k101e (W)*		
*P152L_k101n (W)*		

Mutants are named with the cancer mutation appearing first with capital letters followed by the putative cancer rescue mutation(s) appearing after the underscore. P152L means that the proline at the 152^nd^ amino acid location in p53 is mutated into a leucine. The mutants appearing italicized with a (W), e.g., P152L_k101e, etc., are weak cancer rescue mutants. All others are strong cancer rescue mutants. Artifactual Mutants are cancer rescue mutants that contained more than one cancer rescue mutation or were not in any of the regions, due to background mutagenesis and limitations in early versions of the saturation mutagenesis technique.

**Table 6:** Novel Positive cancer rescue mutants by cancer mutation.

Cancer Mutation	Positive	Negative	Non-MIP
	*(96–105)*	*(223–232)*	*(222–231)*
7: R249S	0	0	0
8: G245S	0	(1)	(1)
14: H179R*	0	0	0
16: R273L	0	0	0
22: R248L	0	0	0
23: R158L	4	(1)	(1)
26: R280T*	0	0	0
27: P151S*	0	0	0
32: P152L*	4+(3)	0	0
34: P278L*	0	0	0
**Total**	8+(3)	0+(2)	0+(2)

The number listed before the cancer mutant is the frequency rank of that cancer mutant occurring in human cancer. e.g., R249S is the 7^th^ most frequent single codon p53 mutation found in human cancer biopsies [12]. Weak Positive cancer rescue mutant counts are in parentheses. Mutants marked with asterisks had never been rescued at the beginning of this study.

### Other Predicted p53 Regions

In addition to Additive Curiosity, six other (non-Random) active learning methods were considered. Table 7 shows the Positive, Negative, and non-MIP regions selected by those other methods. The non-MIP region was similar to the Negative region due to the preponderance of predicted Negative mutants in the test set.

**Table 7:** Region selection by active learning algorithms.

Active Learning Type	Positive	Negative	non-MIP
**Additive Curiosity**	96–105	223–232	222–231
**Maximum Curiosity**	100–109	222–231	222–231
**Minimum Marginal Hyperplane**	141–150	108–117	122–131
**Additive Bayesian Surprise**	96–105	222–231	222–231
**Maximum Entropy**	243–252	206–215	206–215
**Minimum Entropy**	170–179	140–149	140–149
**Maximum Marginal Hyperplane**	210–219	241–250	241–250

The range of numbers listed below the region names are the amino acid locations covered by that region. Note that Minimum Entropy and Maximum Marginal Hyperplane were control active learning methods designed to work particularly poorly. More details are available in the supporting information Table S1.

Minimum Entropy and Maximum Marginal Hyperplane are versions of Maximum Entropy and Minimum Marginal Hyperplane (repectively) designed to do poorly, as negative controls. Indeed, the Negative Region chosen by Minimum Entropy overlaps nine of ten residues with the Positive Region chosen by Minimum Marginal Hyperplane. Similarly the Negative Region chosen by Maximum Marginal Hyperplane overlaps eight of ten residues with the Positive Region chosen by Maximum Entropy.

**Figure 5:**
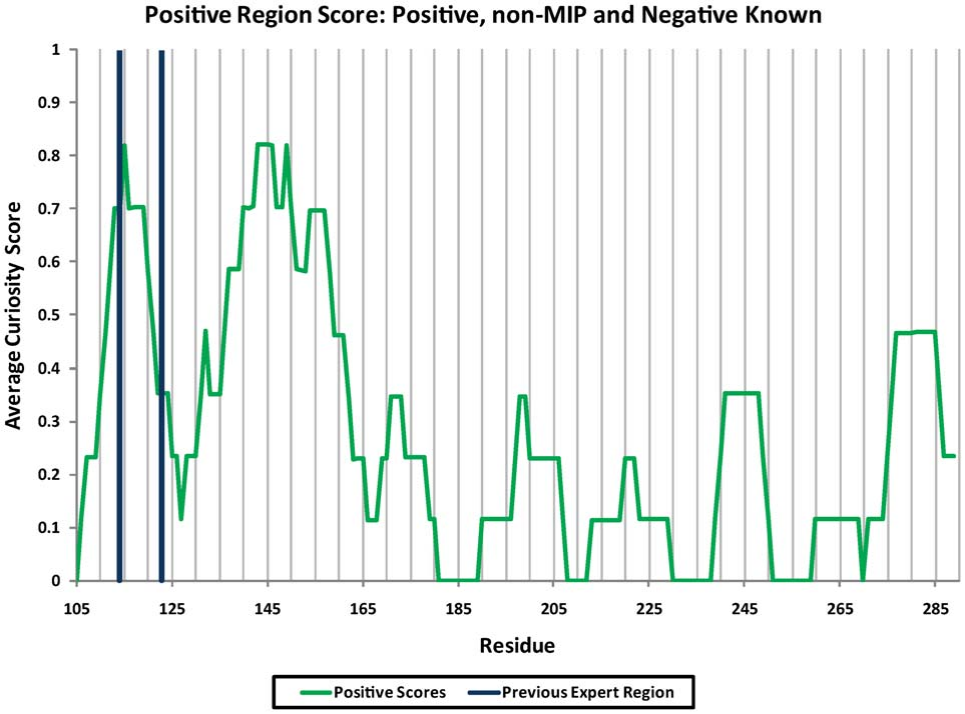
New scores for Positive Regions using new data from the non-expert regions. The score at each residue is the average Additive Curiosity score for those mutants predicted to be Positive for the preceding ten residues. The classifier here was trained with the original 463 mutants used in Figure 4, all Positive cancer rescue mutants found in the Positive, Negative, and non-MIP Regions, and all mutants from those three regions that were not Positive labeled as Negative. The vertical lines show the original Expert Region from residues 114–123.

One might wonder if the classifier would have found the Expert region as a Positive Region in future experiments. Figure 5 indicates the next Positive regions that would be selected, after the mutants found in the current Positive, Negative, and non-MIP regions, but not the Expert region, were added to the training set. There, the most informative positive mutants were found in the region from 130–156, but the region 103–119 also scored well, overlapping the Expert Region (114–123). This is somewhat surprising as the classifier does not consider the Expert criteria, i.e., whether or not this residue appeared in a rescue mutant previously.

### Visualizations of Results

**Figure 6:**
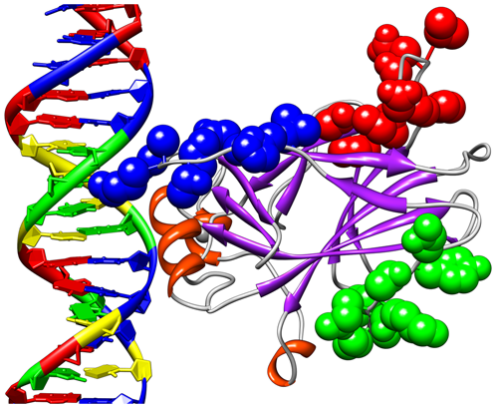
The four p53 regions visualized with the UCSF Chimera package. The blue atoms near the DNA are the Expert Region, the red atoms near the top are the Negative and non-MIP Regions, and the green atoms near the bottom right are the Positive Region.

**Figure 7:**
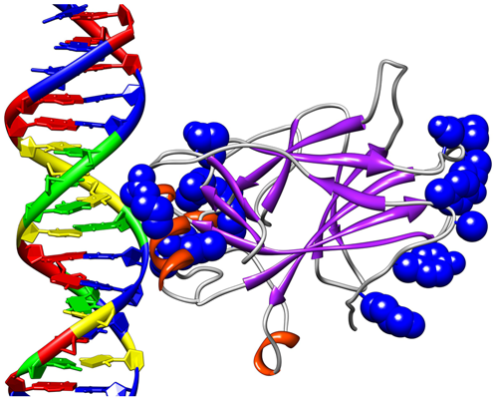
Surface residues selected by mutual information. The blue atoms are those on the p53 surface ranked in the top 50 by the mutual information algorithm as influential on determining classifier accuracy.

**Figure 8:**
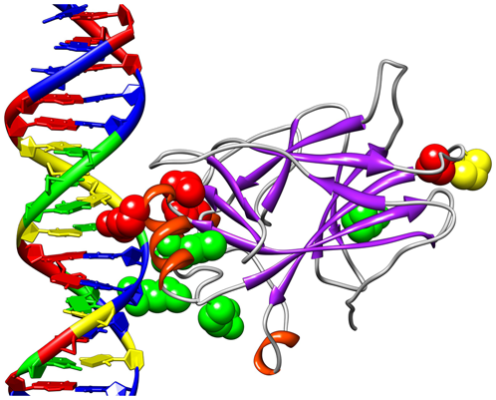
Single cancer rescue mutations. The green atoms clustered mostly in the center of p53 are single amino acid cancer rescue mutations. The blue atoms, such as those on the left and in the lower right corner, are those single cancer rescue mutations that are also selected by mutual information as shown in Figure 7.

**Figure 9:**
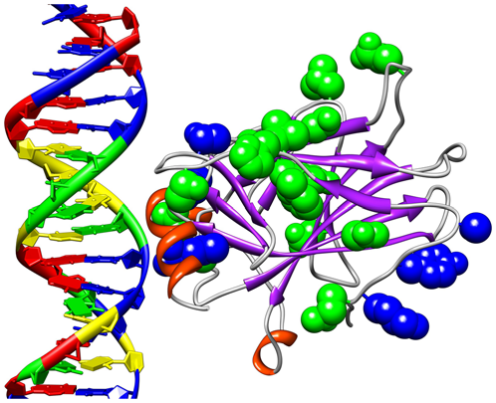
The ten p53 cancer mutants studied here. The red atoms clustered primarily near the top left are those cancer mutations that are currently unrescuable. The green atoms clustered primarily near the lower left are the rescuable cancer mutations. The yellow atoms near the right are the newly rescued P152 mutation.

**Figure 10:**
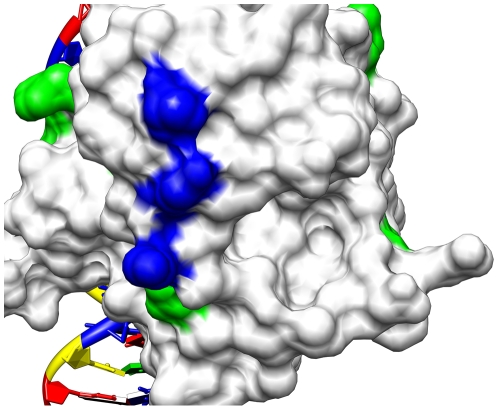
Rescue by p53 surface residues. The above visualization is the same as Figure 9 but rotated and with the surface displayed.

To better understand the regions selected and their relationship to the p53 protein, it is helpful to consider molecular visualizations of p53. Here, p53 is visualized with UCSF Chimera [33],[42]. Figure 6 presents a visualization of the Positive, Negative, non-MIP, and Expert regions on the p53 core domain. It is noteworthy that all of the regions selected in this study appear near the surface of the p53 molecule even though that was not explicitly a criterion in their selection. Figure 7 shows the surface residues selected by the mutual information algorithm [36] to be significant in determining the activity of p53 mutants [12]. Figure 8 shows all known single amino acid rescue mutations. Figure 9 shows the 10 cancer mutants presented in Table 6, Figure 10 including the newly rescued P152L. Figure 10 shows a different visualization of Figure 7.

## Discussion

This paper introduced Most Informative Positive (MIP) active learning, based on machine learning techniques and modeled structure-based features, to help guide biological experiments. The method discovered novel and informative positive results.

### Medical Significance of the Data Set

The ten different cancer mutants studied here account for about one million diagnosed cancers per year. The rescue of cancer mutant P152L by a mutation in the Positive Region is the first report that this common cancer mutant can be rescued at all.

The *in silico* identification and biological verification of a new cancer rescue region is a small but hopefully useful step towards selection of p53 surface regions that potentially result in p53 cancer rescue when appropriately modified. Such regions eventually might be targeted by small molecule drugs. For example, Figure 10 shows an area on the surface of the p53 core domain that is: (1) away from the DNA binding region; (2) overlapping or adjacent to the Positive Region; (3) implicated by mutual information as influential in determining p53 activity; and (4) located where structural changes restore functional activity to some cancerous p53 mutants. Better knowledge of p53 mutant structure-function relationships eventually might lead to successful pharmaceutical manipulation of p53 mutant function.

It has been hypothesized that different p53 cancer rescue mutants have different rescue mechanisms corresponding to different types of cancer mutations [22],[25]. For example, the Expert Region rescued the more frequent p53 cancer mutant G245S while the Positive Region did not. Conversely, the Positive Region is unique in its ability to rescue the P152L mutant. Different rescue regions may implement different rescue mechanisms, and so contribute different facets to knowledge of cancer rescue.

### Extensions

From Figure 4, the Expert Region had both low average curiosity (.462) and relatively few (34) mutants predicted Positive. Thus, this region was not selected by the classifier, yet a significant number of rescue mutants were identified in this region. This is not surprising, as the classifier was not directly exposed to the criteria used for selecting the Expert Region. Conversely, it is not surprising that an expert cancer biologist could pick a fruitful region for reasons unknown to the classifier. Adding expert-level knowledge to a performance system is a long-time success story of artificial intelligence [43]. Integrating diverse expert sources and methods using bioinformatics leads to biomedical discovery acceleration [44]. Adding new features that encode expert or literature knowledge directly into the feature vector that encodes each example is one simple way to make expert knowledge visible to any feature-based learning system.

Similarly, the classifier does not now weigh the medical impact of different p53 cancer mutants. Cancer mutation occurrence frequencies were not given to the classifier, so it is not surprising that it rescued a less frequent cancer mutant than did the expert. Weighting by cancer mutation frequency, or by any other desired utility function, is one simple way to implement a selection preference for some informative Positives over others.

### Conclusion

MIP active learning using modeled structural features was introduced and shown to be a useful framework for function-based biological research. It provided an analysis tool yielding results that otherwise would have been unexpected or unavailable.

From the perspective of a biologist, the computer-selected Positive Region would not have been chosen as a potential region for cancer rescue: It did not contain any known cancer rescue mutants, and none of the random biology-based approaches had ever identified rescue activity in this region. This result provides a proof-of-concept that a computational classifier and modeled structure-based features can provide insight to help guide function-based experimental discovery.

### Availability

All code and data used in this paper is freely available online at https://sourceforge.net/projects/p53cancerrescue/files/. The data is also available in Dataset S1.

All mutant DNA vectors are available under standard material transfer agreements through the UCI Office of Technology Alliances (http://www.ota.uci.edu/).

## Supporting Information

Dataset S1 The raw curiosity scores used to generate Figures 3 & 5 and select the regions shown Table 7.
(2.17 MB ZIP)

Table S1 Table 7 - Region Selection by Active Learning Algorithms as dynamically generated by Microsoft Excel. Intended for use with Dataset S1.
(0.23 MB XLS)

Text S1 Active Learning Related Symbols
(0.06 MB DOC)
