# Supplementary material for: Predicting Positive p53 Cancer Rescue Regions Using Most Informative Positive (MIP) Active Learning
Source: PLoS Comput Biol. 2009 Sep 4;5(9):e1000498. doi: 10.1371/journal.pcbi.1000498 (PMC2742196; doi:10.1371/journal.pcbi.1000498)
Supplement: Text S1 — Active Learning Related Symbols (0.06 MB DOC) [file pcbi.1000498.s003.doc]

# Text S1: Active Learning Related Symbols

$a$: In the case of p53 cancer rescue mutants, $a$ indicates the amino acid number at which certain mutations occur.

$A(m)$: The activity label for a data point $m$. For a binary class, it is convenient to call these labels “Positive” and “Negative”.

$h(m)$: The activity label for a data point $m$ as predicted by the computational classifier.

Halfway Point: The smallest number of iterations $j$ such that $T_{K,j+1}$ contains half of all Positive mutants in $T_{U,1}$.

$i$: The current active learning iteration. $i=1$ during the initial experiment.

$m$: An attribute vector describing each data point.

$n$: The number of data points chosen during each $i$ to become $T_{C,i}$ during *in silico* experimentation.

$score(m)$: A function that assigns a score for ranking data points in $T_{U,i}$.

$T$: The set of all data points under consideration, both the labeled and unlabeled examples.

$T_{C,i}$: The initial subset of data points chosen from $T_{U,i}$ to be labeled.

$T_{K,i}$: The subset of $T$ containing all labeled (known) data points at the $i$th iteration.

$T_{U,i}$: The initial subset of $T$ containing all unlabeled (unknown) data points.

$|T_{X,i}|$: The number of data points in data in set $T_{X,i}$. $|T_{C,i}|=n$.
